# Cognitive biases in first-episode psychosis with and without attention-deficit/hyperactivity disorder

**DOI:** 10.3389/fpsyg.2023.1127535

**Published:** 2023-07-05

**Authors:** Vanessa Sanchez-Gistau, Angel Cabezas, Nuria Manzanares, Montse Sole, Lia Corral, Elisabet Vilella, Alfonso Gutierrez-Zotes

**Affiliations:** ^1^Hospital Universitari Institut Pere Mata of Reus, Reus, Spain; ^2^Institut d’Investigació Sanitària Pere Virgili (IISPV- CERCA), Tarragona, Spain; ^3^Centro de Investigación Biomédica en Red de Salud Mental (CIBERSAM), ISCIII, Madrid, Spain; ^4^Universitat Rovira i Virgili (URV), Reus, Spain

**Keywords:** cognitive bias, CBQp, ADHD, first episode psychosis (FEP), schizophrenia, social cognition

## Abstract

**Introduction:**

Psychotic disorders such schizophrenia and attention-deficit/hyperactivity disorder (ADHD) are neurodevelopmental disorders with social cognitive deficits. Specifically, biased interpretation of social information can result in interpersonal difficulties. Cognitive biases are prevalent in psychosis, but no previous study has investigated whether the type and severity of cognitive biases differ between subjects experiencing first-episode psychosis (FEP) with (FEP-ADHD^+^) and without ADHD (FEP-ADHD^−^).

**Methods:**

A total of 121 FEP outpatients at the Early Intervention Service of Reus were screened for childhood ADHD through the Diagnostic Interview for ADHD (DIVA). Cognitive biases were assessed by the Cognitive Biases Questionnaire for Psychosis (CBQp). CBQp scores of FEPs groups were compared with those of healthy controls (HCs) with an analysis of covariance. Spearman correlation analysis explored associations between CBQp scores and psychopathology.

**Results:**

Thirty-one FEPs met the criteria for childhood ADHD and reported significantly more cognitive bias [median (interquartile range): 47 (38–56)] than FEP-ADHD^−^ [42 (37–48)] and HCs [38 (35.5–43)]. CBQp scores did not differ between FEP-ADHD-and HCs when adjusted for age and sex. After controlling for clinical differences, Intentionalising (*F* = 20.97; *p* < 0.001) and Emotional Reasoning biases (*F* = 4.17; *p* = 0.04) were more strongly associated with FEP-ADHD^+^ than FEP-ADHD^−^. Cognitive biases were significantly correlated with positive psychotic symptoms in both groups but only with depressive symptoms in FEP-ADHD^−^ (*r* = 0.258; *p* = 0.03) and with poor functioning in FEP-ADHD^+^ (*r* = −0.504; *p* = 0.003).

**Conclusion:**

Cognitive bias severity increased from HCs to FEP-ADHD-patients to FEP-ADHD^+^ patients. FEP-ADHD^+^ patients may be a particularly vulnerable group in which metacognitive targeted interventions are needed.

## Introduction

The cognitive model of psychosis suggests that psychotic symptoms may arise because of biased information processing (Garety et al., 2007). In accordance with this model, people with sub threshold (Livet et al., 2020) and full psychotic symptoms (Freeman et al., 2001) are more prone to cognitive biases. Cognitive biases refer to automatic errors in both cognitive processing and content across specific situations (Beck, 1963). A substantial body of research has demonstrated that cognitive biases contribute to the processes of reasoning and metacognition (Garety et al., 2001; Freeman, 2007; Morrison et al., 2007; Bob et al., 2016). From this perspective, the development and maintenance of delusions may be due to the presence of dysfunctional patterns of thought that leads to incorrect judgments and abnormal interpretations or perceptions. The cognitive biases in psychosis that have been most extensively studied are jumping to conclusions (JTC) (Ross et al., 2015; Dudley et al., 2016; McLean et al., 2017) attributional biases (Langdon et al., 2010; Sanford and Woodward, 2017), and belief inflexibility (Moritz and Woodward, 2006). However, it is evident that subjects with psychotic disorder present varied cognitive biases (De Rossi and Georgiades, 2022) including Beck’s emotional biases (Beck, 1963). Thus, the emotional biases of catastrophising (C) and dichotomous thinking (DT) have also been involved in psychoses (Gawęda and Prochwicz, 2015). Peters E and colleagues developed the Cognitive Biases Questionnaire for Psychosis (CBQp) (Peters et al., 2014) to easily and comfortably assess cognitive biases in psychosis. The CBQp was based on the Blackburn Cognitive Styles Test (Blackburn et al., 1986) which was designed to assess frequent cognitive biases in depression. The cognitive biases included in the CBQp are jumping to conclusions (JTC), dichotomous thinking (DT), intentionalising (Int), emotional reasoning (ER), and catastrophizing (C).

Attention-deficit/hyperactivity disorder (ADHD) is a neurodevelopmental disorder affecting 3–7% of school-age children (Polanczyk et al., 2015). It is characterized by motor hyperactivity and impulsiveness and inattention or distractibility that produces functioning problems in the family and school environments’ and in the relationship with peers; frequently, these difficulties persist in adulthood (Barnett, 2016). In addition to impairments in cognitive function, deficits in social cognition and interpersonal difficulties are also important features of ADHD. Within social cognition, deficits in theory of mind and emotion recognition and processing are the domains that have been most investigated; however, findings have been ambiguous (Morellini et al., 2022). Therefore, research on cognitive distortions in ADHD remains scarce, with some studies focusing on attentional and attribution bias (Hartmann et al., 2020; Jenness et al., 2021).

In addition to the genetic overlap between some risk alleles (Hamshere et al., 2013), psychotic disorders and ADHD share some clinical manifestations. Males are overrepresented, both have a high comorbidity with substance abuse, and both manifest difficulties in emotional regulation and peer relationships. Deficits in cognition are central symptoms of neurodevelopmental disorders and have been associated with poor functional outcomes and poor response to treatment. A previous report by our group (Sanchez-Gistau et al., 2020) compared cognitive performance between patients in their first episode of psychosis (FEP) with and without childhood ADHD (c-ADHD) and healthy controls (HCs); we found a gradient in the severity of cognitive impairment, with FEP patients with ADHD (FEP-ADHD^+^ patients) being the most impaired. Compared to FEP-ADHD^−^, FEP-ADHD^+^ were more frequently men, showed a worse antipsychotic response and had a higher risk of drug consumption. The present study builds on this previous study by aiming to determine whether the type and severity of different cognitive biases (measured with the CBQp) differ between FEP patients with and without c-ADHD relative to a control group. In addition, we aimed to investigate the relationship between cognitive bias and psychopathological symptoms in both FEP-ADHD^+^ and FEP-ADHD-patients.

## Methods

### Participants

We invited all consecutive outpatients referred to the Early Intervention Programme (EIP) at the University Hospital Institut Pere Mata of Reus, Spain, from January 2015 to July 2019 fulfilling the following inclusion criteria: age between 14 and 35 years; FEP, defined as the *“onset of full psychotic symptoms within the last 12 months”*; and less than 6 months of antipsychotic treatment. The exclusion criteria were as follows: psychosis induced by substances or other medical conditions, intellectual disability, severe head injury or a lack of fluency in Spanish. During the target period, 152 FEP subjects were referred to the EIP. Six subjects refused to participate and 15 did not fulfil the inclusion criteria. Of the 133 FEP subjects included in our previous study, 11 did not complete the CBQp. Therefore, the final sample consisted of 122 FEP subjects, 31 FEP-ADHD^+^ subjects and 91 FEP-ADHD^−^subjects. The sample of HCs (*N* = 26) was drawn from our previous validation study of the CBQp in the Spanish language (Corral et al., 2020).

Ethical approval was obtained by the Committee for Ethical Clinical and Pharmacological Investigation of the Pere Virgili Research Institute (IISPV). After a complete description of the study was given to the subjects, written informed consent was obtained.

### Assessments

#### Clinical assessments

Clinical assessments were administered by two experienced psychiatrists of the team. Clinical variables related to psychosis, such as the duration of untreated psychosis, current pharmacological treatment, and frequency of drug use in the past 6 months, were assessed through a direct interview. The dose of each antipsychotic was converted to chlorpromazine (CPMZ) equivalents in mg/day (Gardner et al., 2010). We defined as drug users those individuals who used a specific drug *“at least several times a week*.” The severity of psychotic symptoms was assessed using the Positive and Negative Syndrome Scale (PANSS) (Kay et al., 1990) and the severity of affective symptoms by the Calgary Depression Scale for Schizophrenia (CDSS) (Addington et al., 1993) and the Young Mania Rating scale (YMRS) (Young et al., 1978). Finally the level of functioning was assessed by the Global Assessment of Functioning (GAF) (American Psychiatric Association, 1994).

The Spanish version of the Structured Clinical Interview for Diagnostic and Statistical Manual of Mental Disorders, 4th edition (DSM-IV) for Axis I disorders (SCID-I) (First et al., 1997) confirmed the diagnosis of psychosis following DSM criteria. For descriptive purposes we grouped the diagnoses of schizophrenia, schizophreniform and schizoaffective disorders as “schizophrenia spectrum disorders”; manic and depressive episodes with psychotic symptoms as “affective psychoses” and brief psychotic disorders and psychosis not otherwise specified were categorized as “other psychosis.”

#### Assessment of ADHD

A child and adolescent psychiatrist blind to clinical assessments administered the Spanish version of the Diagnostic Interview for ADHD in Adults (DIVA) (Ramos-Quiroga et al., 2019). Additional information was provided by a parent or close relative. The DIVA is the gold-standard assessment for ADHD in adults assessing the severity of each of the 18 symptoms required to meet the DSM-IV diagnostic criteria for ADHD in both childhood and adulthood. Those symptoms must cause impairment in at least two settings and must not be better explained by another psychiatric disorder. The diagnosis is considered definite when six or more criteria are met for each of the symptom domains of hyperactivity-impulsivity and/or attention deficit. We specifically asked about childhood-onset ADHD (c-ADHD) in order to avoid confusion with recent prodromal or current full psychotic symptoms. Adult-onset symptoms were therefore not considered for ADHD diagnoses. FEP subjects fulfilling the criteria for a definite diagnosis of c-ADHD were categorized as FEP-ADHD^+^ otherwise; they were categorized as FEP-ADHD^−^.

#### Assessment of cognitive biases

The CBQp (Peters et al., 2014) has been recently translated and validated for the Spanish population by our group (Corral et al., 2020). It is a self-report questionnaire containing 30 scenarios, 15 involving the theme of Anomalous Perception (AP) and 15 involving the theme of Threatening Events (TE); for each vignette, the subject is asked to choose one of three options that best describe that situation. Six vignettes are included for each of the five cognitive biases: Intentionalising (Int), Catastrophising (C), Dichotomous thinking (DT), Jumping to conclusions (JTC) and Emotional reasoning (ER). The score of each cognitive bias subscale ranges from 5 to 18, and the CBQp total score ranges from 30 to 90 points.

### Statistical analyses

Demographic data were compared among the FEP-ADHD^+^, FEP-ADHD-and HC groups using the chi-squared test with Yates’ correction or Fisher’s exact test for discrete variables and one-way analysis of variance (ANOVA) followed by *post hoc* Tukey pairwise comparisons. Differences in clinical variables between the two FEP groups were explored by the chi-squared test with Yates’ correction or Fisher’s exact test for discrete variables and Student’s t test or the Mann–Whitney U test for continuous variables.

First, differences in cognitive bias (total scores, theme scores and bias scores) between the three groups were analysed by between-subject univariate analysis of variance (ANOVA) followed by *post hoc* Tukey pairwise comparisons. Significant group differences (at *p* < 0.05) were controlled by age and sex by subsequent univariate analysis of covariance (ANCOVA).

Second, differences in cognitive bias scores between clinical groups were analysed while controlling for the effects of clinical variables on which FEP-ADHD^+^ and FEP-ADHD-groups differed at *p* < 0.10. Finally, Spearman correlation analyses were used to separately explore associations between the CBQp total score, TE and SA theme scores, cognitive bias scores and psychopathological symptoms in each clinical group.

All analyses were conducted using IBM SPSS for Windows, version 20.0 (IBM Corp., Armonk, NY).

## Results

### Sociodemographic and clinical characteristics

Thirty-one FEP subjects (25.4%) fulfilled the criteria for c-ADHD: 35.4% as the inattentive subtype, 25.8% as the hyperactive–impulsive subtype, and 38.8% as the combined subtype. Ten out of 31 ADHD subjects (32.25%) had been previously diagnosed with c-ADHD in a child and adolescent mental health unit, but only three were taking treatment for ADHD: one was on methylphenidate, one on guanfacine and one on atomoxetine.

As it can be seen in Table 1, the three groups significantly differed in age and sex. Both clinical groups were significantly younger than the HC group, and males were overrepresented in the FEP-ADHD^+^ group (90.3%). The severity of clinical variables did not differ between the FEP groups, but FEP-ADHD^+^ subjects used tobacco and cannabis more frequently and were treated with a higher dose and a greater number of antipsychotics than FEP-ADHD^−^subjects.

**Table 1:** Socio-demographic and clinical characteristics of the groups.

	FEP Sample (*N* = 122)	FEP-ADHD^+^ (*N* = 31) (A)	FEP-ADHD^−^ (*N* = 91) (B)	Controls *N* = 26 (C)	Statistic (*χ*^2^, *t*)	*p*	*Post hoc*
Socio demographic variables							
Age, years (Mean, SD)	22.2 (5.4)	22.1 (4.8)	22.2 (5.61)	33.4 (6.5)	42.14	<0.001	A = B A < C^***^B < C^***^
Sex (*N*, % of male)	80 (65.6)	28 (90.3)	52 (57.1)	13 (50)	12.9	0.002	A > B^**^A < C^**^B = C
Education years (Median, IQR) Premorbid IQ	10 (8.5–13) 99.4 (15.6)	9 (8–10) 95.2 (14.9)	10 (9–13) 100.2 (16.1)		-1.82^a^ −1.39	0.06 0.16	
Clinical variables							
DUP (Median, IQR)	25 (10–60)	30 (15–87)	21 (10–60)		0.67^a^	0.47	
Diagnoses (*n*, %)					2.744	0.254	
Schizophrenia spectrum disorders Affective psychoses Other psychoses	64 (52.2) 26 (21.3) 32 (26.2)	20 (64.5) 4 (12.9) 7 (22.6)	44 (48.4) 22 (24.2) 25 (27.5)				
PANSS (Median, IQR)							
Positive Negative General total	12 (9–18) 16.5 (11–26) 32.5 (25–44.5) 63 (48–84.5)	13 (8.5–18) 17 (11.5–26.5) 30 (23.5–44) 55 (45.5–92.0)	12 (9–18) 16 (10.5–26) 33 (26–45.5) 64 (48.5–81.5)		0.27^a^ 0.15^a^ −0.29^a^ 0.19^a^	0.78 0.87 0.77 0.84	
GAF (Mean, SD)	57.60 (10.7)	59.7 (11.7)	56.9 (10.3)		1.23	0.21	
CDSS (Median, IQR)	1 (0–6)	1 (0–6.5)	1 (0–6)		0.83^a^	0.40	
YMRS (Median, IQR)	1 (0–9)	2 (0–13)	0 (0–9)		0.41 ^a^	0.68	
Drug abuse (*n*, %)							
tobacco	67 (54.9)	25 (80.6)	42 (46.2)		9.76	0.002	
cannabis	23 (18.9)	11 (35.5)	12 (13.2)		6.12	0.010	
alcohol	23 (18.9)	9 (29.0)	14 (15.4)		1.99	0.16	
Treatment (*n*, %)							
Antipsychotics Number of APs (Median, IQR) CPZE (mg/day) (Median, IQR)	115 (94.3) 1 (1–1) 300 (200–442.4)	30 (96.8) 1 (1–2) 399 (200–600)	85 (93.4) 1 (1–1) 300 (200–400)		0.06 3.05^a^ 1.71^a^	0.80 0.002 0.08	
Antidepressants	28 (23.0)	5 (16.1)	23 (25.3)		0.63	0.42	
Mood stabilizers	21 (17.2)	4 (12.9)	17 (18.7)		0.21	0.64	
Benzodiazepines	44 (36.1)	9 (29.0)	35 (38.5)		0.53	0.46	

SD, standard deviation; IQR, interquartile range; FEP, first episode of psychosis; ADHD, attention-deficit/hyperactivity disorder; DUP, duration of untreated psychoses; IQ, intellectual coefficient; PANSS, positive and negative syndrome scale; CDSS, calgary depression scale for schizophrenia; YMRS, young mania rating scale; GAF, global assessment of functioning; AP, antipsychotic treatment; CPZE, estimated equivalent amount of chlorpromazine.

a Mann-Whitney *U* test. *p < 0.05; **p < 0.01; ***p < 0.001.

### Differences in cognitive biases

As shown in Table 2, the ANOVA revealed group differences in all cognitive biases except for JTC. *Post hoc* pairwise comparisons showed that FEP-ADHD^+^ patients scored significantly higher than HCs on all scores. After adjusting for age and sex, these differences remained significant except for the C bias. FEP-ADHD-patients exhibited significantly higher scores than HCs on the CBQp total score, the TE theme and the ER bias; however, after adjusting for age and sex, these differences were no longer significant.

**Table 2:** Comparison of cognitive biases between the groups.

				ANOVA results				ANCOVA ^a^results			
CBQp (median/IQR)	FEP-ADHD+ *N* = 31 (A)	FEP-ADHD- *N* = 91 (B)	Controls *N* = 26 (HC)	F (p) *Post-ho*c Between subjects				F (p) *Post-hoc* Between subjects			
CBQ total	47 (38–56)	42 (37–48)	38 (35.7–43)	9.11^***^	A > B^*^	A > C^***^	B > C^*^	5.88^*^	A > B^**^	A > C^**^	B = C
Themes											
TE (Threatening events)	24 (21–32)	22 (19–25)	20 (18–22)	8.23^***^	A > B^*^	A > C^***^	B > C^*^	4.31^**^	A > B^*^	A > C^**^	B = C
AP (Anomalous perception)	23 (18–26)	20 (18–23)	18 (17–20)	7.44^**^	A > B^*^	A > C^**^	B = C	5.85^***^	A > B^**^	A > C^**^	
Biases											
Int (Intentionalising)	9 (7–12)	8 (6–8)	7 (6–8)	17.33^***^	A > B^***^	A > C^***^	B = C	12.61^***^	A > B^***^	A > C^**^	
C (Catastrophism)	9 (8–11)	8 (7–10)	8 (7–9)	3.38^*^	A = B	A > C^*^	B = C	2.76			
DT (Dichotomous thinking)	10 (8–12)	8 (7–10)	7 (7–8)	8.86^***^	A > B^*^	A > C^*^	**B = C**	4.40^**^	A > B^*^	A > C^**^	
JTC (Jumping to conclusions)	9 (9–11)	9 (8–11)	9 (9–10)	0.83				0.91			
ER (Emotional reasoning)	9 (7–11)	8 (7–10)	7 (6–8)	7.72^**^	A > B^*^	A > C^***^	B > C^*^	2.80^*^	A > B^*^	A > C^**^	B = C

IQR, interquartile range; FEP, first episode of psychosis; ADHD, attention-deficit/hyperactivity disorder; HC, healthy control; A, PEP-ADHD^+^; B, PEP-ADHD^−^; C, healthy controls.

*p < 0.05; **p < 0.01; ***p < 0.001.

^a^Differences between 3 groups adjusting for sex and age.

The two clinical groups were further directly compared after adjusting for sociodemographic and clinical differences at a threshold of *p* < 0.10, that is, after adjusting for sex, years of education, tobacco and cannabis use and antipsychotic dose (See Table 3). Compared to FEP-ADHD-patients, FEP-ADHD^+^ patients scored significantly higher on the CBQp total score (*F* = 7.11; *p* = 0.009), TE (*F* = 4.10; *p* = 0.04) and AP (*F* = 8.94; *p* = 0.003) themes and Int (*F* = 20.97; *p* < 0.001) and RE (*F* = 4.17; *p* = 0.04) biases.

**Table 3:** Comparison of CBQ between FEP ADHD+ and FEP-ADHD-.

Cognitive Biases (median, IQR)	FEP sample (*N* = 122)	FEP-ADHD^+^ (*N* = 31)	FEP-ADHD^−^ (*N* = 91)	ANCOVA^a^ Statistic *p*	
CBQp Total	43 (37.5–50.5)	47 (38–56)	42 (37–48)	7.11	0.009
Themes					
TE (Threatening Events)	22 (20–26)	24 (21–32)	22 (19–25)	4.10	0.04
AP (Anomalous Perception)	20 (18–24)	23 (18–26)	20 (18–23)	8.94	0.003
Biases					
Int (Intentionalising)	8 (7–9)	9 (7–12)	8 (6–8)	20.97	<0.001
C (Catastrophism)	8 (7–10)	9 (8–11)	8 (7–10)	2.95	0.10
DT (Dichotomous thinking)	9 (7–10)	10 (8–12)	8 (7–10)	2.53	0.11
JTC (Jumping to conclusions)	9 (8–11)	9 (9–11)	9 (8–11)	0.34	0.56
ER (Emotional reasoning)	8 (7–10)	9 (7–11)	8 (7–10)	4.17	0.04

IQR, interquartile range; CBQp, cognitive biases questionnaire for psychosis; IQR, interquartile range.

FEP, first episode of psychosis; ADHD, attention-deficit/hyperactivity disorder.

a adjusted by: sex, years of education, cannabis and tobacco use and CPZE estimated equivalent amount of chlorpromazine dose.

### Correlation between psychopathological symptoms and cognitive biases

In the correlation analyses (Table 4), the CBQp total score was significantly correlated with the PANSS positive symptoms subscale score (PANSS-P) in both clinical groups and was correlated with the PANSS general symptoms subscale score (PANSS-G) and CDSS score in only the FEP-ADHD^−^ group. In the FEP-ADHD^+^ group, the CBQp total score was also correlated with lower GAF scores. Regarding the TE and AP themes, TE was correlated with the PANSS-P scores in both clinical groups and with the PANSS-G and CDSS scores in the FEP-ADHD^−^ group. The AP theme score was associated with PANSS-P and PANSS-G scores in only the FEP-ADHD^−^ group.

**Table 4:** Association of cognitive biases with psychopathological symptoms and daily functioning in the clinical groups.

	FEP-ADHD^+^ (*N* = 31)						FEP-ADHD^−^ (*N* = 91)					
	PANSS-P	PANSS-N	PANSS-G	CDSS	YMRS	GAF	PANSS-P	PANSS-N	PANSS-G	CDSS	YMRS	GAF
CBQt	0.410^*^	0.202	0.253	0.225	0.167	−0.504^**^	0.379^**^	0.092	0.260^**^	0.258^*^	0.207	−0.188
CBQ-TE	0.468^*^	0.232	0.357	0.248	0.171	−0.214	0.273^*^	0.023	0.186	0.257^*^	0.070	- 0.157
CBQ-AP	0.259	0.181	0.117	0.135	0.140	−0.390^*^	0.433^**^	0.184	0.293^**^	0.159	0.207	−0.183
CBQ-Int	0.208	0.157	0.072	0.158	0.003	−0.338^*^	0.236^*^	0.030	0.049	0.057	0.149	0.037
CBQ-C	0.415^*^	0.276	0.291	0.162	0.284	−0.306	0.211^*^	0.062	0.163	0.216^*^	0.133	−0.086
CBQ-DT	0.294^*^	0.004	0.251	0.226	−0.079	−0.243	0.377^**^	0.045	0.284^**^	0.364^**^	0.109	−0.265^*^
CBQ-JTC	0.444^*^	0.190	0.214	0.152	0.136	−0.458	0.196	0.089	0.226^*^	0.292^**^	0.052	−0.230^*^
CBQ-ER	0.334	0.286	0.260	0.098	0.328	−0.238	0.357^**^	0.158	0.272^*^	0.135	0.144	−0.129

Spearman correlation coefficients (r); *p value < 0.05; **p value < 0.01.

FEP, first episode of psychosis; ADHD, attention-deficit/hyperactivity disorder; HC, healthy control; PANSS, positive and negative syndrome scale; PANSS-P, PANSS positive symptom subscale; PANSS-N, PANSS negative symptom subscale; PANSS-G, PANSS general symptom subscale; CDSS, calgary depression scale for schizophrenia; YMRS, young mania rating scale; GAF, global assessment of functioning; CBQp, cognitive biases questionnaire for psychosis; CBQt, CBQp total score; CBQ-TE, CBQp threatening events (TE) theme; CBQ-AP, CBQp anomalous perceptions (AP) theme; CBQ-I, CBQp intentionalising (I) subscore; CBQ-C, CBQp catastrophising (C) subscore; CBQ-DT, CBQp dichotomous thinking (DT) subscore; CBQ-JTC, CBQp jumping to conclusions (JTC) subscore; CBQ-ER, CBQp emotional reasoning (ER) subscore.

Regarding cognitive biases, Int was positively correlated with the PANSS-P score in FEP-ADHD-patients and with worse GAF scores in FEP-ADHD^+^ patients. Positive correlations were found between the C and DT bias scores and the PANSS-P score in both clinical groups and between the CDSS score in the FEP-ADHD^−^ group. The DT bias score also correlated with the PANSS-G score in the FEP-ADHD^−^ group. JTC correlated with PANSS-P score in FEP-ADHD^+^ patients and with general PANSS-G scores, and CDSS score in FEP-ADHD-patients. Finally, the ER bias score was associated with positive PANSS-P and PANSS-G scores in only in the FEP-ADHD^−^ group.

## Discussion

As far as we know, this is the first study to assess and compare the severity of cognitive bias (measured by the CBQp) between FEP-ADHD^+^ and FEP-ADHD^−^ subjects relative to HCs. Our results therefore must be considered preliminary and caution is required when interpreting the subsequent findings.

We found a gradient of cognitive bias severity from the FEP-ADHD^+^ group: [median (interquartile range) 47 (38–56)], to the FEP-ADHD^−^ group [42 (37–48)] to the HC group [38 (35.5–43)]. The FEP-ADHD^+^ group presented significantly higher scores not only on the total score but also in the CBQp themes of TE and AP than both the FEP-ADHD-and HC groups. Regarding specific cognitive biases, FEP-ADHD^+^ patients exhibited greater Int, DT, C and ER bias scores than HCs, while FEP-ADHD-patients presented only greater ER bias scores than HCs. The FEP-ADHD^+^ group therefore showed the most marked differences from HCs. However, the HC group used as a control group was not specifically recruited for the present study but was used in our previous study to validate the questionnaire in the Spanish population (Corral et al., 2020). Consequently, groups differed in terms of age and sex ratios. When the results were further adjusted for these differences, differences between FEP-ADHD-patients and HCs were no longer significant. In contrast, FEP-ADHD^+^ patients still significantly scored higher than HCs, except for the C bias score. Unexpectedly, the three groups did not significantly differ in the JTC bias score. JTC is the most investigated cognitive bias in psychosis, and when assessed by the probabilistic behavior task (the beads task) (Garety et al., 2005), JTC bias has been frequently observed in patients with established schizophrenia (Ross et al., 2015; Dudley et al., 2016; McLean et al., 2017) and FEP (Falcone et al., 2015) as well as in those at clinical risk of psychosis (Livet et al., 2020). However, when assessed by self-report questionnaires, mixed results have been reported (Bastiaens et al., 2013; Ahuir et al., 2021; Pena-Garijo et al., 2022; Pugliese et al., 2022). One possible explanation is that patients with psychosis often have a little awareness of their cognitive deficits and biases (Moritz et al., 2004). Therefore, a dissociation between the objective (assessed by task performance) and subjective measures (assessed by self-report) cannot be ruled out. In addition, Beck’s cognitive biases have an emotional component rather than a psychotic cognitive–perceptual component, which may explain why they are also present in the healthy population (Bastiaens et al., 2018). Unfortunately, depressive and anxiety symptoms were not evaluated in the HC group. Thus, it would have been useful in order to identify a potential relationship between the emotional component and the cognitive distortions in that group.

A novel finding of the present study is that FEP-ADHD^+^ patients showed more severe cognitive biases than FEP-ADHD-patients, even after controlling for clinical and sociodemographic differences. Apart from scoring higher on the two themes (AP and TE), it is particularly interesting that the FEP-ADHD^+^ group exhibited more DT and significantly more Int and ER than the FEP-ADHD^−^ group. Consistent with these findings, in their original validation report comparing subjects with psychosis with HCs and depressed subjects, Peters and colleagues (Peters et al., 2014) reported that the psychosis group scored higher than the depressed group on the Int and ER biases. Moreover, Int was the only bias where the depressed group and HCs did not score differently. The authors suggested that these two specific biases may represent a particular “paranoid thinking style” that distinguishes individuals with psychosis from other clinical populations. In accordance, we found that FEP-ADHD^+^ patients were more likely to exhibit the Int bias (*F* = 20.97; *p* < 0.001). The Int bias refers to the implicit and automatic inclination to interpret human actions as intentional and to think that negative actions toward oneself were committed on purpose (i.e., intentionally). In the ADHD literature, research on interpretation bias is very scarce. There is inconsistent evidence that hostile attribution bias (HAB) with ambiguous situations and ambiguous faces occurs more frequently in children and adults with ADHD than in HCs (King et al., 2009; Sibley et al., 2010; Schneidt et al., 2019). No previous study to date has addressed this issue in FEP patients with and without ADHD; thus, further investigation is needed to replicate or refute our results and disentangle whether FEP patients with c-ADHD exhibit greater cognitive bias than FEP patients without c-ADHD.

Notably, when exploring the relationship between cognitive biases and psychopathological symptoms, only weak correlations were found. Regarding specific biases, the severity of positive symptoms was associated with the C and DT biases in both clinical groups; however, the severity of positive symptoms was associated with the JTC bias in the FEP-ADHD^+^ group and with the Int and ER biases in the FEP-ADHD^−^ group. Nevertheless, positive correlations between cognitive biases and depressive symptoms and general symptoms were found only in FEP-ADHD-patients. Although neither clinical group differed in psychopathological symptoms or functioning, our study suggests a different pattern of biases related to positive, general and depressive symptoms in FEP-ADHD^+^ patients compared to FEP-ADHD-patients. Moreover, poor functioning was associated with the CBQp total score and the Int bias in only the FEP-ADHD^+^ group. Given the lack of previous studies, we can only speculate that the dominant cognitive biases in FEP-ADHD-patients may be related to depressive and anxious symptoms. On the other hand, the severity, type of cognitive biases and the lack of relationship with depressive and general symptoms in the FEP-ADHD^+^ group may reflect traditional psychotic thinking, which in turn might be associated with worse functioning. Moreover, children with ADHD present deficits in recognizing facial emotions and others’ emotional states in addition to deficits in emotional processing (Pishyareh et al., 2015). Thus, we speculate that greater cognitive biases in the FEP-ADHD^+^ group may interact with the social processing difficulties already present in ADHD to pose a higher risk of impaired functioning.

Our results extend previous findings suggesting that young adults with c-ADHD and FEP suffer additional impairments (Peralta et al., 2011; Rho et al., 2015; Sanchez-Gistau et al., 2020). Specifically, we report for the first time greater cognitive biases (in general) and more severe Int and ER biases (in particular) in FEP-ADHD^+^ patients. Together, these findings indicate that FEP-ADHD^+^ subjects may be a particularly vulnerable group and a high-priority target for interventions addressing both cognitive biases. Metacognitive training therapy (MCT) was developed by Moritz S and colleagues two decades ago to address problems related to cognitive biases and social cognition in psychosis (Moritz and Woodward, 2007). Previous research has indicated that MCT is an effective psychological intervention for people with schizophrenia (Moritz and Lysaker, 2018; Moritz et al., 2022). Specifically, in patients with recent-onset psychosis, MCT has demonstrated to be effective for improving psychotic symptoms, cognitive insight, and attributional style, as well as for reducing cognitive distortions (Ochoa et al., 2017; Ahuir et al., 2018). Our findings therefore may have clinical relevance for treatment recommendations. As reported, ADHD is a prevalent condition in FEP accompanied by prominent cognitive bias; thus, an adapted intervention for this subgroup aiming to reduce the most prevalent bias can be recommended. Our findings indicate the necessity of conducting metacognitive intervention studies specifically designed to assess the effectiveness of these particular interventions in this particular subgroup of patients.

### Limitations and strengths

Some limitations must be taken into account when interpreting our finding. With regards the ADHD diagnoses, despite we used an structured interview for assessing ADHD symptoms recall bias regarding childhood onset symptoms cannot be entirely ruled out. We tried to avoid the possibility of overlapping ADHD symptoms with psychotic symptoms by restricting the diagnoses of ADHD to childhood-onset, that is, onset of symptoms before the age of 7 years according to the DSM-IV criteria. Moreover, the healthy control group HCs who participated in the previous validation study was not specifically assessed for ADHD and differed in terms of age and sex distribution. Second, despite controlling for clinical and socio-demographic differences, the scores of CBQp might have been influenced by other variables that were not adjusted for, such as variables related to stress and childhood trauma. It has also to be acknowledged that sample size limited our ability to conduct secondary analyses stratified by ADHD subtype or by psychotic diagnoses. Moreover, the low percentage of females prevented us to investigate sex differences in the studied variables and ADHD.

However, despite these limitations, we have included a real-world clinical practice sample in their early stages of the illness coming from a particular geographical area. Our relatively homogeneous sample, allows us to minimize the impact of the burden of a chronic disease and long-term antipsychotic treatment. Finally cross-sectional assessment did not allow us to infer a causal relationship between cognitive bias and ADHD in FEP patients.

In summary, we report a gradient of severity in CBQp scores among the three groups, with the FEP-ADHD^+^ group differing the most markedly from the FEP-ADHD-and HC groups. The severity of cognitive biases, however, did not differ between the FEP-ADHD-and HC groups after adjusting for age and sex. Importantly, the Int and ER biases were the most strongly associated with the FEP-ADHD^+^ group, but no bias was associated with the FEP-ADHD^−^ group.

## Conclusion

Our present findings together with previous findings indicate that FEP-ADHD^+^ subjects represent a clinical subgroup with a worse potential prognosis than FEP-ADHD^−^ subjects. Further research on the relationships among cognitive biases, cognitive performance and environmental factors are needed to develop individualized pharmacological and psychological interventions, such as MCT, for FEP subpopulations. The relationship between ADHD and psychosis is still an important knowledge gap that requires further investigation.

## Data availability statement

The data that support the findings of this study are available from the corresponding author upon a reasonable request.

## Ethics statement

The studies involving human participants were reviewed and approved by the Committee for Ethical Clinical and Pharmacological Investigation of the Pere Virgili Research Institute (CEIM of IISPV). Written informed consent to participate in this study was provided by the participants’ legal guardian/next of kin.

## Author contributions

VS-G designed the current study. AC, MS, VS-G, and LC contributed to the acquisition of clinical data. NM was blinded to the results of the clinical assessments and performed the ADHD interviews. VS-G, AG-Z, and EV undertook the statistical analysis and all authors took part in the interpretation of the results. VS-G wrote the first draft of the article. All authors critically revised the first draft and provided their contributions and have approved the final manuscript.

## Funding

This work was supported by the AGAUR Agència de Gestió d’Ajuts Universitaris i de Recerca (SGR2017_444).

## Conflict of interest

The authors declare that the research was conducted in the absence of any commercial or financial relationships that could be construed as a potential conflict of interest.

## Publisher’s note

All claims expressed in this article are solely those of the authors and do not necessarily represent those of their affiliated organizations, or those of the publisher, the editors and the reviewers. Any product that may be evaluated in this article, or claim that may be made by its manufacturer, is not guaranteed or endorsed by the publisher.
